# The experiences of bedside nurses delivering an intensive care sedation study: A process evaluation within the A2B trial

**DOI:** 10.1177/17511437251381951

**Published:** 2025-11-06

**Authors:** Lydia M. Emerson, Bronagh Blackwood, Kalliopi Kydonaki, Cathrine McKenzie, Timothy S. Walsh, Leanne M. Aitken

**Affiliations:** 1 School of Health & Medical Sciences, City St George’s, University of London, UK; 2 The Medical School, University of Exeter, UK; 3 School of Medicine, Dentistry and Biomedical Sciences, Queens University Belfast, UK; 4 Faculty of Nursing, National and Kapodistrian, University of Athens, Greece; 5 School of Medicine, University of Southampton, NIHR Biomedical Research Centre Southampton, Perioperative and Critical Care Theme, Department of Pharmacy and Critical Care, University Hospital Southampton, UK; 6 Dept of Anaesthesia, Critical Care and Pain Medicine, Usher Institute, University of Edinburgh, UK

**Keywords:** qualitative research, critical care, process evaluation, nurses, sedation

## Abstract

**Background:**

This process evaluation explored delivery of a complex sedation intervention within the Alpha-2 Agonists for Sedation to Produce Better Outcomes from Critical Illness (A2B) trial, which compared dexmedetomidine- and clonidine-based sedation with propofol (usual care). All groups targeted lighter sedation levels. The objective was to understand bedside nurses’ experiences delivering the interventions and identify factors influencing protocol adherence and implementation.

**Methods:**

A qualitative study using semi-structured interviews was conducted with intensive care unit (ICU) staff (consultants, bedside and research nurses) from A2B trial sites. Thematic analysis explored how participants experienced and delivered trial interventions, with particular focus on bedside nurses’ abilities to manage sedation in line with the protocol.

**Results:**

Nurses with greater ICU experience described more confidence and adaptability in using alpha-2 agonists, while less experienced staff required support due to limited familiarity with lighter sedation. Hesitancy to up-titrate alpha-2 agonists was common, driven by concerns about bradycardia and hypotension. Reluctance to down-titrate propofol was shaped by fears of agitation and self-extubation. Deep sedation norms, especially amongst nurses trained during the COVID-19 pandemic, further hindered protocol adherence. Research nurses were instrumental in supporting implementation and bridging knowledge gaps.

**Conclusion:**

Despite all three trial groups targeting lighter sedation, nurse confidence, safety concerns, and ingrained cultural practices limited adherence to alpha-2 agonist-based protocols. Addressing these barriers through training, support, and cultural change will be essential for future trials and practice shifts involving lighter sedation strategies in ICU.

**Trial registration number:**

ClinicalTrials.gov NCT03653832 https://clinicaltrials.gov/study/NCT03653832

## Background

Sedation management in ICUs is essential for patient safety and comfort, allowing critically ill patients to tolerate mechanical ventilation, minimise discomfort, and avoid agitation. Traditionally, ICU sedation has favoured deeper sedation to ensure ventilator compliance and reduce risks like agitation or accidental self-extubation, although recent evidence challenges this paradigm.^1^ Deep sedation, while effective in the short term, is linked to poorer outcomes such as prolonged mechanical ventilation and ICU stays. Evidence suggests lighter sedation strategies may improve outcomes but could pose risks to long-term psychological well-being.^2,3^ Propofol, typically used with intravenous (IV) opioid infusions, is recommended as first-line sedation in clinical guidelines. The alpha-2 agonist dexmedetomidine is also being increasingly used due to its capacity for lighter sedation and analgesic properties that might reduce opioid use.^4^ Clonidine is an alpha-2-agonist used widely in the UK, instead of dexmedetomidine. A 1-day point prevalence of UK ICU sedation practices reported 10.4% of ventilated patients were receiving dexmedetomidine, as opposed to 5.5% receiving clonidine.^5^

The Alpha-2 Agonists for Sedation to Produce Better Outcomes from Critical Illness (A2B) trial compared dexmedetomidine and clonidine to standard propofol sedation. The primary outcome was time to successful extubation, with secondary outcomes including ICU mortality, time to discharge, sedation quality, delirium rates, and cardiovascular events. Patients were randomised into three groups (propofol, dexmedetomidine, clonidine) accompanied by an IV opioid infusion, with additional pain relief as needed, and continued propofol permitted in the alpha-2 agonist groups where necessary. The goal across all groups was to maintain light sedation (Richmond Agitation-Sedation Scale (RASS) of −2 to +1) meaning patients were calm but easily rousable. For alpha-2 agonist groups, rapidly increasing trial medication post-randomisation was crucial, aiming for sedation primarily via the allocated drug.

The trial found no significant difference in time to extubation or sedation quality and delirium measures.^6^ However, significantly higher agitation and bradycardia rates occurred in alpha-2 agonist groups. Continued low-level propofol use (20%–30%) persisted in both intervention groups, and around 25% of patients required deeper sedation during the intervention illustrating the complexity of changing sedation practices within trial conditions.

Nurses are central in ICU sedation protocol implementation, managing titration and monitoring. Their experiences significantly influence intervention feasibility and fidelity. The A2B trial required addressing entrenched practices like overnight deeper sedation or beliefs that deeper sedation is ‘safer’ for avoiding adverse events. However, lighter sedation was accepted as best practice across all trial groups and was not an area of differentiation in the trial protocol. Managing known side-effects of alpha-2 agonists such as bradycardia, hypotension, and ileus remained challenging. Recognising ICU sedation complexity, the A2B trial included a process evaluation to examine factors such as nurse experience, unit culture, resources, and clinical decision-making, essential for optimising sedation protocols and improving outcomes.

The process evaluation was conceptually informed by a framework that adapts elements from existing models for evaluating complex interventions,^7–10^ developed specifically to meet the challenges of research in ICU settings. This approach draws on established guidance while addressing the realities of high-acuity, team-delivered care and organisational variation. It reflects a pragmatic orientation, focussed on understanding how participant- and organisational-related factors shape implementation, delivery, and intervention outcomes.

## Aims and objectives

This study explored bedside nurses’ experiences delivering a complex sedation intervention within the A2B trial, aiming to understand contextual factors affecting implementation, protocol adherence, and sedation-related decision-making. Key areas included the influence of nurse experience on intervention delivery, reluctance to up-titrate alpha-2 agonists, and hesitation to reduce or discontinue propofol. The evaluation also considered broader challenges in shifting from deep to lighter sedation practices, shaped by entrenched cultural norms and nursing apprehensions. The COVID-19 pandemic, which occurred during the trial, may have amplified these issues.^11^ Increased reliance on deep sedation to manage ventilated patients, alongside staff shortages, reinforced these practices – leaving a lasting impact on nurses’ confidence with lighter sedation strategies in the post-pandemic period.^12^

## Methods

### Design

This paper reports the qualitative component of a mixed-methods, multi-phase process evaluation, the methods of which have been reported elsewhere.^13^ The focus was on exploring the experiences of bedside nurses in delivering a complex sedation intervention as part of the A2B trial. Following Medical Research Council (MRC) guidance,^10^ the process evaluation examined how the intervention was implemented and the influence of nurse experience on its delivery. The process evaluation was informed by the MRC framework for complex interventions, particularly its emphasis on implementation, context, and mechanisms of impact. Recognising the distinct challenges of delivering and evaluating interventions in critical care, such as high clinical acuity, multidisciplinary decision-making, and organisational variability, we used an adapted process evaluation approach developed specifically for use in intensive care and perioperative trials. This framework builds on established models to support the exploration of how complex interventions are implemented and experienced in time-pressured, team-based environments, while remaining grounded in core process evaluation concepts, including intervention fidelity, dose, reach, and context.

Ethical approval for both the main A2B trial and the embedded process evaluation was granted by the South Central – Oxford C Research Ethics Committee (REC reference: 19/SC/0351). All participants in the qualitative component provided written informed consent prior to participation.

The A2B intervention comprised multiple interacting components including pharmacological, behavioural, and organisational elements, and was designed with both fixed and flexible components. Fixed elements included the use of a target sedation range (RASS -2 to +1), implementation of bedside titration protocols, and the requirement to use the allocated sedative as the primary agent. Flexible elements allowed for local adaptation, including unit-specific education and support strategies, and variation in bedside research nurse support, and clinician discretion on temporary propofol continuation if clinically justified. This complexity, and its influence on intervention delivery and trial outcomes, is explored in greater detail in the full process evaluation report.^14^

The intervention was implemented across a diverse range of ICUs that varied in their prior research experience, staffing models, and cultural norms related to sedation. Some sites had substantial research infrastructure, experience in sedation trials, and higher research nurse availability, while others operated with more limited resources and had less exposure to complex trial protocols. Cultural attitudes towards sedation also varied, with some units maintaining deeper sedation practices—particularly overnight—due to staffing limitations or perceptions of safety. These contextual differences influenced both fidelity to the intervention and how trial protocols were adapted in practice. Further details are presented in the full process evaluation report.^14^

A logic model mapping inputs, processes, and expected outcomes outlined the A2B intervention and its underlying assumptions. The evaluation centred on validating these assumptions through in-depth interviews with ICU staff, with particular emphasis on bedside nurses’ perspectives. The logic model was developed during trial design and used throughout the process evaluation to guide data collection and analysis. While the model itself was not revised, its assumptions were examined using implementation outcomes such as fidelity, dose, reach, and contextual adaptability, as described in this manuscript and in the full process evaluation report.^14^

While the wider process evaluation explored the perspectives of the full multidisciplinary team, this paper specifically examines experiences and views relating to bedside nurses and their delivery of the intervention. This includes both the perspectives of bedside nurses themselves and those of other staff who supported or observed intervention delivery. This focus reflects the central role of bedside nurses in implementing the sedation protocol and the pivotal influence they had on fidelity and local adaptation. Interviews explored practical and emotional challenges in protocol adherence, alpha-2 agonist titration, and managing sedation adjustments in high-pressure environments. An extensive pre-trial exploration of UK ICUs ensured contextual relevance, identifying factors such as unit culture, staffing, and sedation practices influencing intervention delivery. Insights from this pre-trial work highlighted potential barriers and facilitators specific to bedside nurses. Crucially, the evaluation also accounted for the contextual challenges of delivering lighter sedation practices within ICUs that were recovering from the strain of the COVID-19 pandemic, given the aim was to target light sedation in all trial groups consistent with best practice.^15^ The pandemic led to significant shifts in ICU staffing patterns, including accelerated onboarding of less experienced nurses and widespread deep sedation.^11^ These pandemic-driven changes were considered integral to understanding how the A2B trial interventions were implemented and the extent to which nurses adapted to the lighter sedation protocols. This contextual focus provided nuanced insights into protocol adherence, decision-making, and factors shaping A2B trial implementation across diverse ICU settings.

### Data collection and analysis

Semi-structured interviews occurred involving ICU staff integral to the trial (Principal Investigators (PIs), research nurses, bedside nurses, ICU consultants). Interviews and analyses were conducted by an experienced qualitative researcher with extensive expertise in critical care process evaluations. The interview guide, developed by the process evaluation team, was designed to explore intervention implementation and delivery based on the trial’s logic model. It was reviewed and refined in consultation with the A2B Trial Management Group and co-investigator team, which included clinicians (intensivists, anaesthetists, pharmacists, nurses), methodologists, and a PPI representative (see Supplementary Material).

A core process evaluation team of five members worked collaboratively on data analysis throughout the study, contributing to validation, interpretation, and ensuring analytical rigour. To support this, a second researcher with clinical and methodological expertise reviewed a sample of transcripts and coding. While transcripts were not returned to participants due to the clinical context, confirmatory responses were used during interviews to ensure accurate understanding of participant perspectives. Reflexivity was maintained through regular team discussions, documentation of interpretive decisions, and cross-review of transcripts and coding. The process evaluation was conducted independently from trial delivery, enabling critical distance while drawing on the team’s combined methodological and clinical expertise.

Phase 1 interviews began after 3 months of patient recruitment using purposive sampling based on recruitment rates, usual sedation practices, research nurse resources, and unit size. Interviews assessed implementation influences and intervention fidelity. Phase 2 interviews occurred during the trial’s final 6 months, limited to units actively recruiting within the 3 months prior, optimising recall. These interviews focussed on organisational and participant-related factors affecting intervention delivery, adherence to the trial protocol, medications, decision-making, and pandemic-related staffing impacts.

**Figure 1.**
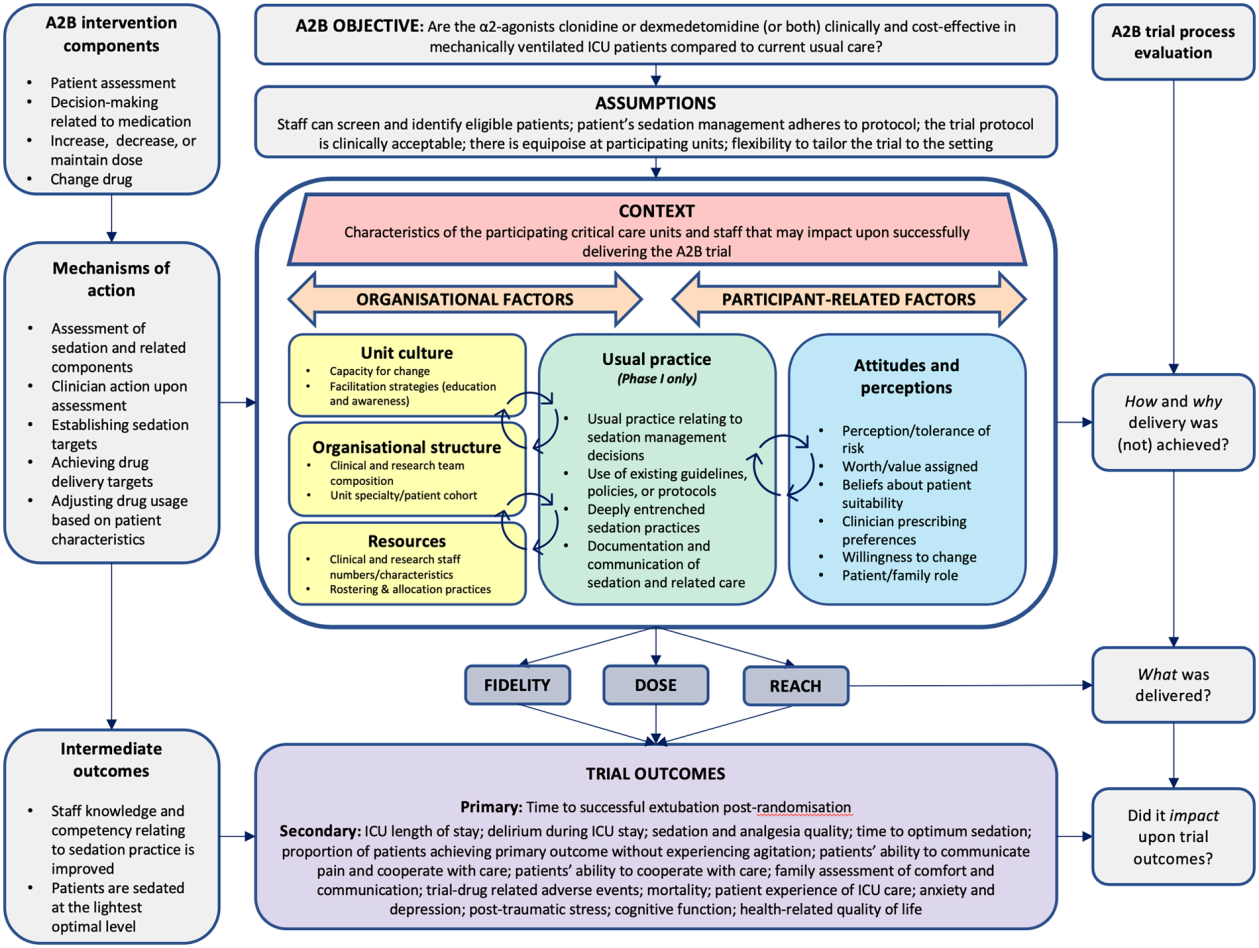
A2B trial logic model.

Interviews via Microsoft Teams lasted 45–60 min, were securely recorded, and transcribed verbatim. Semi-structured guides informed by the logic model^13^ (Figure 1) ensured consistent exploration across diverse ICU settings. Interviews were analysed using a seven-step Framework approach,^16^ involving deductive coding guided by the logic model (developed pragmatically by the research team to reflect the specific context of the trial), with inductive examination of unallocated data. Themes were iteratively refined through team consensus. The Framework approach provided structured, transparent analysis, maintaining interpretive rigour. Synthesised qualitative findings clarified implementation challenges, providing critical insights into trial delivery and contextual factors.

This framework analysis was guided by a pragmatic, context-sensitive framework developed specifically to address the complexities of critical care intervention delivery and evaluation. While the approach facilitated deductive coding aligned with the trial’s logic model, it also allowed for inductive exploration of data that did not fit the initial framework, ensuring analytic flexibility. This balanced method supported rigorous, credible findings relevant to the ICU setting.

## Results

Phase 1 interviews (January–April 2023) and Phase 2 (September–December 2023) each included 12 purposively sampled ICUs selected from 30 active trial sites. Sites were purposively selected using a four-criteria sampling matrix (research nurse provision – including whether nurses were ICU-trained; unit size; routine use of sedation targets; and the site’s red-amber-green recruitment rating) to ensure spread across contexts. Within selected sites, volunteers were invited from key professional roles (research nurses, bedside nurses, PIs, and other relevant staff), seeking representation across grades and more than one participant per group across the sample. Sample size was not fixed a priori; recruitment continued until the matrix strata and role/grade representation were populated within trial timelines. Sixty-nine staff were interviewed across both phases: 40 research nurses, 10 bedside nurses, 18 PIs, and 1 clinical trials assistant.

Thematic analysis identified key factors influencing sedation delivery. Nurse experience and hierarchy were central, shaping adherence to the protocol. Experienced nurses exhibited greater confidence and autonomy, while less experienced nurses often deferred to senior colleagues. Hesitancy in up-titrating alpha-2 agonists stemmed from side-effect concerns (e.g., bradycardia, hypotension) and reluctance to deviate from familiar sedation approaches. Perceived unpredictability of alpha-2 agonists intensified this caution. Down-titrating or discontinuing propofol was also challenging, driven by safety concerns and staffing, particularly overnight when senior support was reduced.

### How did nurse experience impact intervention delivery?

Experience of bedside nurses, including duration of ICU experience and specific experience of administering trial medications, played a critical role in the successful delivery of the intervention, particularly when managing patients randomised to alpha-2 agonists. These medications, while occasionally used in routine practice, were not typically considered first-line sedatives. This posed significant challenges for less experienced nurses, who often required additional bedside support and education to adhere to the protocol. Less experienced nurses struggled with the demands of titrating or modifying alpha-2 agonists, as their limited exposure to these agents in comparable clinical contexts contributed to hesitancy and a reliance on more experienced staff or medical input for decision-making. The crux of their reluctance stemmed from the unknown: they were unfamiliar with the drug’s speed of action, perceived unpredictability, and potential side effects. Importantly, this reluctance was not rooted in uncertainty about dosing, as the bedside algorithms were consistently cited as helpful and clear. Instead, it was the lack of practical experience and confidence with managing these agents in real-time clinical situations that amplified their reliance on senior staff and contributed to delays or deviations from protocol adherence. By contrast, more experienced nurses described a higher level of confidence in managing alpha-2 agonists in alignment with the trial protocol. Their extensive clinical backgrounds, shaped by years of exposure to a variety of sedative agents, enabled them to independently assess and adjust sedation levels. These experienced nurses were better equipped to navigate the unpredictable and complex situations often encountered in ICU. Their familiarity with protocols and sedative practice allowed them to address potential side effects, such as bradycardia or hypotension, with greater assurance—barriers that less experienced nurses often perceived as insurmountable. Both clinical staff and research nurses consistently emphasised the strong correlation between the experience of bedside nurses and their ability to deliver the intervention effectively.

Experienced nurses not only exhibited greater confidence but also played a pivotal role in optimising adherence to the protocol. Discussions often highlighted how, even in cases where experienced nurses had limited prior exposure to using alpha-2 agonists in the specific manner required by the trial, their broader ICU expertise allowed them to adapt and manage challenges proactively. Research nurses noted that experienced staff were more likely to adhere to the protocol independently, requiring less intensive oversight compared to their less experienced counterparts. For less experienced nurses, the circumstances of their entry into ICU challenged adherence to the protocol. Many had joined ICUs during or after the COVID-19 pandemic—a time marked by the widespread normalisation of deep sedation practices. These nurses often had no prior experience managing ventilated patients who were more awake or independently titrating sedation levels, as such practices had largely been side-lined during the pandemic. Consequently, less experienced staff tended to default to maintaining deeper sedation levels, which were more familiar and perceived as safer. This reliance on established but outdated approaches to sedation management created significant barriers to adapting to the trial’s emphasis on lighter sedation and the down-titration of propofol. Hierarchical decision-making further influenced these dynamics. Research nurses noted that less experienced nurses often deferred to more experienced colleagues or medical staff when faced with uncertainty, even in straightforward scenarios. This reliance on hierarchy, while understandable given their limited experience, delayed timely adjustments to sedation and perpetuated existing barriers to adherence. Conversely, more experienced staff demonstrated greater autonomy and adaptability, which streamlined protocol delivery.

“. . .*so experience meant that if they weren’t familiar with the drug, they were happy enough for us to say, look, it’s fine, stick it on, and they knew they’d be able to cope with whatever happened after that, so they stuck it on. Experience either gave them the confidence to deal with something new or it meant that that wasn’t a new thing, they’d done it loads of times before.*” ResN16/04

“. . .*they’ve got a million and one things to do, we [research nurses] set the drug up for them, we’ll stay until it’s established a decent rate, so I think we just don’t want them to be left thinking, oh my God, what am I doing, cause I think most of us remember what it’s like being that bedside nurse. So we just want to make sure that they’re comfortable, because then we get the best out of them by adhering to the protocol, isn’t it?*” ResN05/02

### Why were alpha-2 agonists not more proactively up-titrated?

Bedside nurses frequently hesitated to up-titrate alpha-2 agonists in accordance with the protocol due to concerns about potential side effects, including cardiovascular (bradycardia, hypotension), gastro-intestinal (ileus), and a broader concern relating to the slower onset of sedation compared to the rapid effect of propofol. Many nurses expressed heightened anxiety when patients’ heart rates approached the low sixties, fearing a rapid progression to severe bradycardia. This hypervigilance often led to pre-emptive decisions to hold or reduce the doses of alpha-2 agonists, even when the protocol indicated that a continuation or increase was appropriate. These concerns were compounded by a perception that alpha-2 agonists carried an inherent unpredictability, further heightening reluctance to adjust doses upward without explicit guidance. Similar findings have been reported in prior studies, where the perceived unpredictability of alpha-2 agonists discouraged dose escalation, despite protocol guidance.^17^ Research nurses noted that this cautious approach was more pronounced for patients enrolled in the trial compared to routine clinical practice. It was frequently reported that bradycardia did not elicit the same level of concern in non-trial settings, suggesting that nurses perceived an additional layer of accountability and scrutiny when administering alpha-2 agonists as part of the trial. This increased sense of responsibility often led to overly conservative practices, as staff sought to avoid potential complications that could arise under trial conditions. The hierarchical nature of ICU decision-making further reinforced this reluctance. Less experienced nurses were particularly hesitant to up-titrate alpha-2 agonist medications without explicit instructions from experienced staff or medical colleagues. This deference to hierarchy often resulted in delays or omissions in up-titrating doses, with less experienced staff expressing concern about accountability for adverse events. Research nurses played a key role in mitigating these delays by providing direct support and reassurance, yet their absence overnight and on weekends left gaps in adherence.

“*But we did get a lot of, oh that’s the one that caused that patient to have. . .to go brady or. . .we did get a lot of, oh but A2B caused that, that’s. . .that trial caused that. . . I found that, you know, every time you went up then, the first. . .next few patients, I would have got a lot of, oh but he’s already sitting at 60, you know, he can’t really afford to go. And it. . .the. . .it doesn’t always make you. . .you know, it’s. . .it was. . .there was a lot of reluctance after it*.” ResN14/38

“*I think people, kind of, panic as soon as someone becomes a little bit brady as well. And I think because we hammer home about bradycardia, people get a little bit overcautious and stop it. It’s like. . .there’s a balance*.” ResN12/11

### Why did nurses hesitate to down-titrate or discontinue propofol?

Bedside nurses were also reluctant to down-titrate or turn off propofol in alignment with the protocol, primarily due to concerns about patient safety. Many nurses feared that reducing sedation levels would result in patients becoming agitated, unmanageable, or even self-extubating. Although only one incident of self-extubation occurred among the 933 patients randomised to alpha-2 agonists during the trial—and the patient maintained their airway without complications—these fears persisted. This anticipated risk of harm influenced staff decisions to maintain deeper sedation as a protective measure. These fears may be rooted in ICU cultural norms, which favour deeper sedation as a perceived safety measure.^18^ The perception of inadequate staffing further compounded these concerns. Nurses described feeling unprepared to manage potential complications from lighter sedation, particularly during overnight shifts when staffing was reduced, and senior medical support was limited. Overnight shifts were often staffed by junior doctors, who shared similar anxieties about managing crises without immediate access to experienced colleagues. This environment further encouraged the default practice of maintaining deeper sedation levels overnight and maintaining or increasing propofol doses. In addition to safety concerns, the hierarchical structure of decision-making further hindered adherence. Less experienced nurses, who often worked overnight shifts with limited access to experienced colleagues, expressed reluctance to down-titrate propofol without explicit medical direction. This dependency slowed transition to lighter sedation and perpetuated the trial’s challenges in altering established sedation norms.

“*I think the biggest concern from some of the nursing staff was the fact that, you know, if the patient woke up and became agitated, you obviously can’t bolus clonidine, you can’t bolus dexmedetomidine, so it was, like, what do we do, what do we do. And I was, like, well keep some propofol handy, you know, just an ampule of it, or something so that if you need to*. . .” ResN20/17

Site-level barriers were addressed through ongoing collaboration between the trial team and ICU staff. While the process evaluation itself was observational, responsive support was provided during trial delivery to address identified challenges such as staffing pressures, sedation culture, and unfamiliarity with trial drugs. These interactions varied by site and were reflected in differing levels of fidelity, dose, and reach, as described in this manuscript and further explored in Aitken et al. (in press).^14^

## Discussion

**Figure 2.**
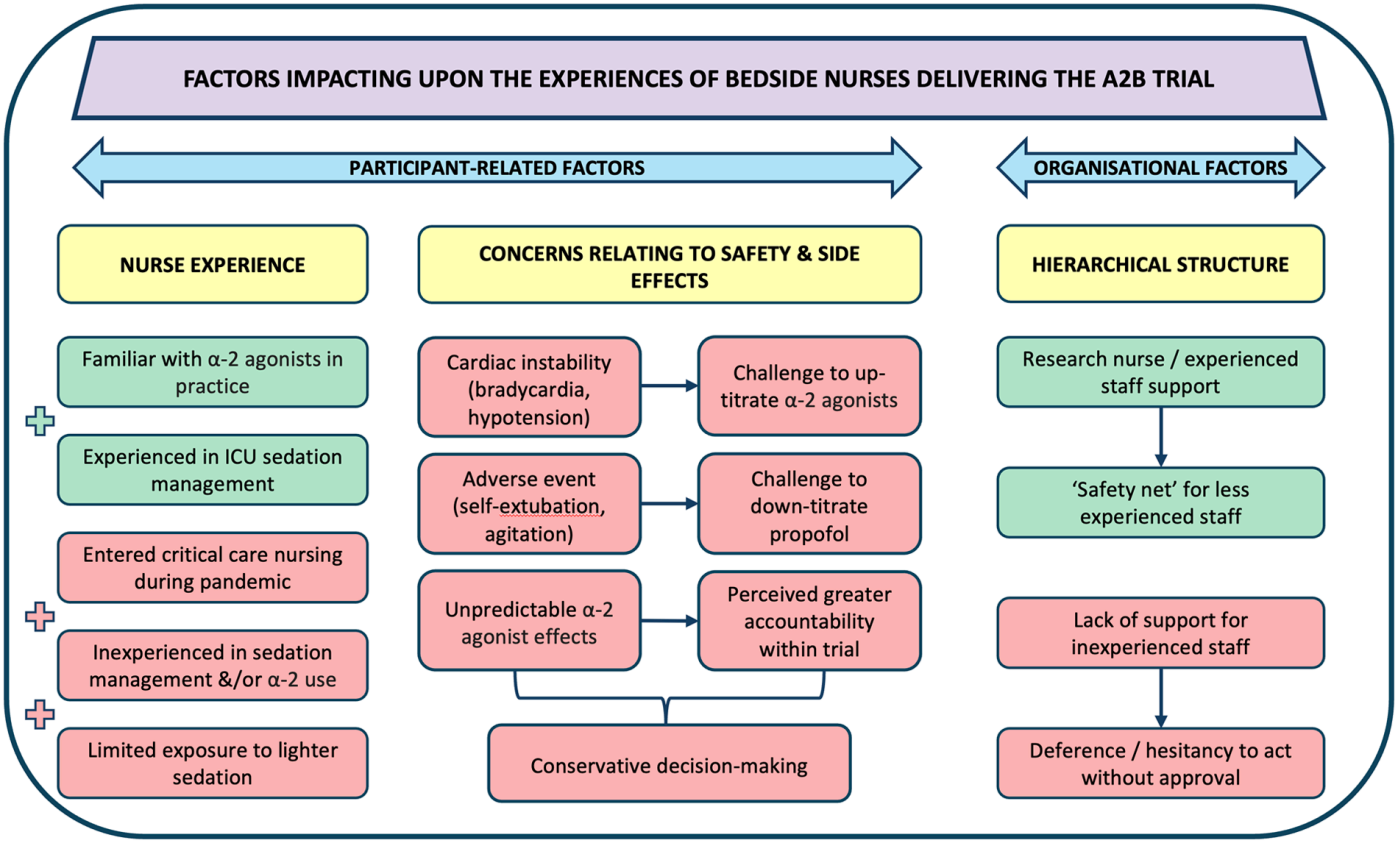
Summary of factors impacting upon the experiences of bedside nurses delivering the A2B trial. (Yellow boxes indicate the overarching themes in the discussion. Green boxes represent facilitating factors under that theme. Red boxes represent barriers under each theme).

When a complex intervention trial like A2B does not demonstrate a measurable effect on outcomes, it is important to explore possible contributing factors. This process evaluation sought to identify such factors focussing on bedside nurses’ experiences. While the intervention aimed to achieve sedation primarily based on alpha-2 agonists with minimal propofol use, variation in protocol adherence was observed. Nurse experience (particularly within ICU) and a reliance on hierarchical structure influenced protocol implementation. Safety concerns, including bradycardia and agitation, likely constrained uptake. Nurses frequently hesitated to up-titrate alpha-2 agonists and fully down-titrate propofol, influenced by concerns over adverse effects and limited confidence in using these agents. The frequent continuation of low-dose propofol use (20–30% of rates in the propofol group) reflects these implementation challenges. The patient population exhibited typical ICU heterogeneity consistent with real-world clinical settings. However, incomplete implementation, dosing variability, and local adaptations based on clinical preference may have reduced the potential impact of the intervention. These findings point to contextual and implementation barriers (including experience, hierarchy, and ICU culture) that influenced the trial results and may limit translation of evidence-based sedation strategies into routine care. These are summarised in Figure 2. Contextual variation between sites also likely contributed to implementation differences. ICUs differed in their baseline sedation practices, organisational support for research, and experience with complex trial protocols. These site-level differences shaped how the intervention was received, how readily it was adapted into practice, and the degree of staff engagement. Aitken et al. (in press) explores these variations in depth and highlights their impact on fidelity and local adaptation.^14^

### Nurse experience and its impact on intervention delivery

Nurse experience, both general ICU and with trial medications, appeared to influence how successfully the intervention was delivered. Experienced nurses often described greater confidence and independence, which may have supported better adherence. Their background helped them feel confident to manage side effects and adjust sedation levels, which aligns with evidence suggesting that professional confidence among ICU nurses is a key enabler for adhering to complex sedation protocols.^19^

Professional confidence may support nurses in managing complex interventions more effectively, acting as a mechanism that facilitates role transition, allowing nurses to handle challenging scenarios with greater autonomy and assurance.^20^ This aligns with the findings in this trial, where experienced nurses were better equipped to manage trial protocols independently, contributing to higher fidelity in protocol adherence. Similarly, expertise among ICU nurses is cultivated through years of practice and exposure to high-stakes situations. This expertise may support the ability to anticipate complications, respond to patient-specific needs, and engage with novel interventions such as alpha-2 agonists as first line sedatives.^21^ The ability of experienced nurses to make critical decisions and proactively manage side effects likely contributed to the smoother implementation of the intervention within this trial.

In contrast, less experienced nurses, many commencing their ICU practice during the COVID-19 era of deep sedation, had limited exposure to lighter sedation or alpha-2 agonists. This lack of exposure to trial practices and medications created hesitancy and dependency on more experienced colleagues or medical staff. Consequently, deviations from the protocol were more likely, with less experienced nurses defaulting to familiar but outdated practices. These findings highlight the potential value of considering staff experience when designing interventional trials. To mitigate this, targeted training, simulation, mentorship, and bedside support—such as trial champions—can promote confidence and improve delivery. Mentorship and professional development are particularly valuable in fostering confidence and skill among nurses,^20^ bridging the experience gap and promoting adherence to evidence-based sedation practices.

### Hierarchy as a barrier or facilitator

ICU hierarchy appeared to have mixed effects. On one hand, it offered safety for less experienced nurses, who sought guidance from senior staff, but delayed titration decisions. This hesitancy reflected concerns over accountability. Conversely, hierarchy can also serve as a facilitator when leveraged effectively. Experienced nurses and research staff were described as playing important roles in supporting less experienced colleagues and promoting protocol adherence. For instance, research nurses often set up trial medications and provided bedside reassurance, which helped less experienced nurses build confidence. However, gaps in support, particularly during overnight shifts when senior staff were less available, highlighted the need for more robust systems to mitigate these challenges.

Hierarchy in healthcare organisations has been reported to shape communication, particularly between staff with differing levels of authority or expertise, affecting not only what is said but also who feels comfortable speaking up and under what circumstances. This dynamic may have important implications for how decisions are made in high-pressure environments like the ICU. Hierarchical structures may also reinforce power imbalances, creating barriers to communication and increasing stress for those in less powerful positions.^22^ To maximise the positive aspects of hierarchy while minimising delays in decision-making, future trials should foster a culture that empowers all staff, regardless of seniority or experience, to engage confidently with trial protocols and raise concerns.^23^ Clear communication and a supportive team culture may help less experienced nurses navigate uncertainty and gradually build autonomy. Simulation-based teamwork training and interdisciplinary debriefings can further enhance collaboration and ensure that hierarchical dynamics support, rather than hinder, effective sedation management.

### Addressing proactive up-titration of alpha-2 agonists

Reluctance to up-titrate alpha-2 agonists appeared to stem from concerns about potential side effects, particularly bradycardia and hypotension. Nurses’ heightened anxieties, particularly when patients’ heart rates approached the low sixties, often led to conservative decision-making. Despite protocol guidance, nurses reportedly reduced or withheld doses in some cases. This cautious approach was exacerbated by a perception that alpha-2 agonists were inherently unpredictable and carried greater accountability under trial conditions. ICU nurses’ management of sedation appears to be shaped by concerns about patient safety, previous experiences with medications, and the cultural norms within their units.^19^ These factors may lead nurses to prioritise the avoidance of adverse events over strict protocol adherence, particularly when medications are perceived as risky. This aligns with accounts in this study, where safety concerns about alpha-2 agonists sometimes appeared to take precedence over the intended intervention, despite protocol guidance. A lack of familiarity with alpha-2 agonists also contributed to hesitancy in dose escalation. ICU nurses are more confident managing medications they have used extensively in usual practice.^19,21^ When unfamiliar medications are introduced, nurses may feel less equipped to manage potential complications, which could contribute to more cautious practice.

To help address this hesitancy, future trials may benefit from emphasising education and practical training on the pharmacological properties and safety profiles of trial medications. Providing real-time data on patient outcomes and reinforcing the clinical evidence supporting the use of alpha-2 agonists may help alleviate anxiety and build trust in the intervention. Additionally, fostering a culture of shared accountability, where decisions are made collaboratively with input from the multi-disciplinary team, can reduce individual apprehension and promote timely protocol adherence.^19,21^

### Challenges in down-titrating or discontinuing propofol

Reluctance to reduce propofol appeared to be influenced by safety concerns and longstanding clinical habits. Concerns about agitation or self-extubation, while reportedly rare, continued to shape sedation decisions. These concerns were compounded by staffing limitations, particularly during overnight shifts, where reduced medical support heightened nurses’ reluctance to transition to lighter sedation. ICU norms have been reported to favour deeper sedation, particularly during periods of stress or reduced support.^1^ This was reflected in the current trial, where concerns about agitation or self-extubation appeared to contribute to the continued use of propofol, even when protocols encouraged transitioning to lighter sedation.

Sedation management has also been described as a balancing act between alleviating patient distress and minimising adverse outcomes, which further complicated adherence to lighter sedation protocols.^24^ Limited familiarity with managing awake, ventilated patients, along with concerns about unpredictable response, appeared to contribute to hesitancy, particularly among less experienced nurses. Staffing constraints, especially during overnight shifts, left nurses with limited support, reinforcing a preference for deeper sedation to avoid perceived risks. Previous research suggests that nurses may find managing lighter sedation challenging in ventilated patients, particularly when faced with uncertainty about patient responses and limited access to senior support during critical times. These challenges may be intensified by the high stress and workload typical of ICU settings, which can drive nurses towards deeper sedation practices as a perceived safer and more manageable option.^25^

Cultural resistance to lighter sedation has been widely reported, requiring shifts in both mindset and practice.^26^ To overcome this, trial protocols should include contingency plans for managing potential complications associated with lighter sedation. Adequate staffing levels and access to experienced support during all shifts may help mitigate these concerns. Sharing positive trial outcomes, such as the successful management of lightly sedated patients, can foster confidence and drive cultural change.

This study has a range of strengths and limitations. It highlights the vital role of bedside nurses in clinical trial implementation and intervention delivery, an area often under-explored in ICU research. The process evaluation explored ICU sedation practices during and after COVID-19, addressing a critical and contemporary challenge in ICU. The inclusion of 24 ICUs across two phases provided a broad sample of sedation practices, capturing contextual variations through wide sampling and insights from both research and clinical teams, thereby strengthening the reliability of the findings. Despite these strengths, the study’s confinement to UK ICUs may limit the applicability of the findings to international ICU settings. Further, the collection of the majority of process evaluation data in the 2 years after the COVID-19 pandemic situate it in a specific place in time.

The findings from this evaluation highlight the need for targeted strategies such as the following to optimise trial delivery in ICU settings:

- Enhance Nurse Education and Training – Implement comprehensive education programmes and simulation-based training to familiarise staff with trial protocols and medications. Hands-on practice with alpha-2 agonists in controlled settings can build confidence and competence.
- Leverage Hierarchical Dynamics – Promote a culture of mentorship and collaboration, where experienced staff actively support less experienced colleagues. Structured debriefings and interdisciplinary teamwork training can reinforce positive hierarchical interactions.
- Strengthen Bedside Support – Integrate research nurses more fully into routine care to provide real-time guidance and reinforce protocol adherence, particularly during high-risk periods such as overnight shifts.
- Seek wider multiprofessional support in sedation practice to support bedside nurses
- Address Systemic Constraints – Ensure adequate staffing levels and access to senior medical support during all shifts. Develop clear escalation pathways for managing complications and encourage shared decision-making to reduce individual apprehension.
-  Foster a Culture of Evidence-Based Practice – Highlight the clinical benefits of lighter sedation and alpha-2 agonists through continuous education and feedback. Sharing success stories and positive patient outcomes can help shift cultural norms towards evidence-based sedation practices.

## Conclusion

This study highlights the complex interplay of clinical, cultural, and systemic factors that influenced the delivery of the A2B trial protocol. Key barriers included limited experience among less experienced nurses, safety concerns, hesitancy to titrate alpha-2 agonists and entrenched norms favouring deeper sedation. These challenges, coupled with hierarchical dynamics and staffing constraints, impacted adherence to the trial protocol and may have contributed to the outcomes observed. Collaboration between research nurses and clinical staff proved instrumental in bridging knowledge gaps and supporting protocol adherence. This analysis focuses specifically on nursing roles in delivery; broader multidisciplinary collaboration is explored in the full process evaluation report.^14^ However, the findings underscore the importance of integrating research practices into routine care while fostering an inclusive research culture that empowers all nurses, regardless of seniority, to confidently engage with trial protocols.

## Supplemental Material

sj-docx-1-inc-10.1177_17511437251381951 – Supplemental material for The experiences of bedside nurses delivering an intensive care sedation study: A process evaluation within the A2B trial Supplemental material, sj-docx-1-inc-10.1177_17511437251381951 for The experiences of bedside nurses delivering an intensive care sedation study: A process evaluation within the A2B trial by Lydia M. Emerson, Bronagh Blackwood, Kalliopi Kydonaki, Cathrine McKenzie, Timothy S. Walsh and Leanne M. Aitken in Journal of the Intensive Care Society
