# Supplementary material for: The experiences of bedside nurses delivering an intensive care sedation study: A process evaluation within the A2B trial
Source: J Intensive Care Soc. 2025 Nov 6;27(2):145–54. doi: 10.1177/17511437251381951 (PMC12597800; doi:10.1177/17511437251381951)
Supplement: sj-docx-1-inc-10.1177_17511437251381951 – Supplemental material for The experiences of bedside nurses delivering an intensive care sedation study: A process evaluation within the A2B trial [file sj-docx-1-inc-10.1177_17511437251381951.docx]

**Supplementary Materials 1**

# **The A2B trial investigators**

Chief Investigator: Timothy S Walsh

Lead Statistician: Christopher J Weir

Statisticians: Richard Parker; Sharon Tuck

## **Trial Management Group:**

Timothy S Walsh, Christopher J Weir, Richard Parker, John Norrie, Julia Boyd, Alix MacDonald, Annabel Giddings, Gayle Beveridge, David Hope, Sîan Irvine, Leanne M Aitken, Lydia M Emerson, Cathrine A McKenzie, Nazir I Lone, Kalliopi Kydonaki, Benedict Creagh-Brown, Stephen Morris, Valerie J Page.

## **Grant Co-applicants:**

Leanne M Aitken, Cathrine A McKenzie, John Norrie, Nazir I Lone, Kalliopi Kydonaki, Benedict Creagh-Brown, Stephen Morris, Daniel Francis McAuley, Paul Dark, Matt P Wise, Anthony C Gordon, Gavin Perkins, Michael Reade, Bronagh Blackwood, Alasdair MacLullich, Robert Glen, Valerie J Page, Christopher Weir, Richard Parker.

## **Trial Steering Committee**

Independent Members: Natalie Pattison (Chair), Barry Williams (PPI representative), Louise Rose, Paul Mouncey, John Prowle, David Wellsted

Non-independent members: Timothy S Walsh (CI), Stephen Brett (external advisor), Heather Charles (sponsor representative), Christopher J Weir (Blinded Statistician), John Norrie (Co-applicant)

## **Independent Trial Data Monitoring and Safety Committee**

Julian Bion (Chair), Graeme McLennan, Matt Stevenson, Alistair Nichol

## **Participants at Contributing sites**

**Lothian**

Timothy Walsh (PI), Maria Amamio, Lucy Barclay, Sophie Birch, Kate Briton, Sarah Clark, Jessica Crossan, Katherine Doverman, David Hope, Lucy Macdonald, Corrienne McCulloch, Nicola Rae, Scott Simpson, Jo Singleton, Maggie Wishart, Ruth Thompson, Neill Aitken, Rachel Fairlie, Nabeel Salim, Sam Talbot, Sarah Ackroyd, Valeria Alicino, Euan Allan, Thomas Anderson, Rosemary Andrew, Andrew Baigey, Kenneth Baillie, Calum Barnetson, Ruth Begbie, Richie Biggers, Michael Blaney, Richard Broom, David Birrell, Will Calkin, Keegan Chuavilong, Rebecca Cowden, Thomas Cox, Coral Darjee, Simon Davies, Annemarie Docherty, Luke Dornan, Mark Dunn, Stuart Edwardson, Ross Gillespie, Jane Greenwood, David Griffith, Alasdair Hay, Amy Hu, Ali Hunter, Karen Jones, Helen Jordan, Ancy Joseph, Kallirroi Kefala, Stephanie Kelly, Laura Kemp, Bara Kubanova, Victoria Leng, John Livesey, Nazir Lone, James Lyon, Olivia Mansfield, Dean McAvoy, Aaron McClatchey, Jonathan Miller, Ananda Mirchandani, Peter Moffitt, Steven Morrison, Alexandra Muir, Kieran Nunn, John Ochiltree, Emily Ogden, Matthew Parks, Marc Pass, Rachael Penrose, Harry Putnam, Thomas Quinn, Jonathan Rhodes, Alexander Rollings, Stephen Ross, Ralph Shackleton, Manu Shankar, Sunil Sharma, Iain Slessor, Zack Slevin, Duncan Stickle, Louise Symons, Fiona Walker, Luke Walls, Ian Whiteford, Sue Yin Yong, Neil Young

**Royal Alexandra Hospital**

Kevin Rooney (PI), Michael Kinsella, Brian Digby, Michael Brett, Paul McConnell, Mark Henderson, Radha Sundaram, Lisa Gemmell, Fiona Christie, Philip Henderson, Fiona MacGregor, Steven Henderson, Natalie Rodden, Kirsty Fallon, Lynn Abel, Barbara McLaren, Emma Hughes, Deborah McGlynn, Nicola Thomson, Lauren Walker, Susan Currie, Natasha Parker, Donna Gillan

**North Tees and Hartlepool**

Farooq Brohi (PI), Sarah Purvis, Michele Clark, Pam Race, Lynne Williams, Ahmed Shahin, Eusebius Nworah, Jonathan Gui, Li-Chin Cheng, Katelyn Stewart

**Southampton**

Rebecca Cusack (PI), Mark Tomlin (previous PI), Clare Bolger, Rachel Burnish, Sue Jackson, Alice Baker**,** Jonathan Biss, Karen Salmon, Michael Carter, Catherine McKenzie, Razaz Elsheikh, Missy (Anne) Harrison, Charlotte Thomas, James Ward, Andrew Cumpstey, Ahilanandan Dushianthan, Ivan Kemp

**West Hertfordshire**

Valerie Page (PI), Xiaobei Zhao, Nazril Nordin, Ahmed Hegazy, Elvira Hoxha, Owen Hardaker, Chimenime Ede, Nailia Kotrikova, Acharya Devaraja, Thomas Stambach, Prasun Mukherjee, Mark Louie Guanco

**Cardiff and Vale**

Matthew P Wise (PI), Jade Cole, Helen Hill, Jenny Brooks, Michelle Davies, Rhys Davies, Emma Thomas, Angharad Williams, Lauren Lodhi, Matt PG Morgan

**Countess of Chester**

Simon Ridler (PI), Christopher Smith, Maria Faulkner, Alison Ivison, Laura McKay, Helen Jeffrey, Jude Price, Lucy Slater, Angela Davies, Edward Hughes.

**North Bristol**

Matt Thomas (PI), Dominic Janssen, Ian Thomas, Kate Crewdson, Christopher Newell, Robert Hirst, Stephen West, Agnieszka Skorko, Emma Gendall, Ruth Worner, Beverley Faulkner, Borislava Borislavova, Kati Hayes, Andrew Parsons, Elizabeth Goff, John Sowersby, Annie Wood**,** Kieran Oglesby, Idrisu Sanusi, Charlie Pope, Andrew Baird**,** Hayley Blackmore, Robert Healey

**King’s College**

Philip Hopkins (PI), Eleanor Corcoran, Gillian Selman, Clare Finney, Evita Pappa, John Smith, Emma Clarey, Maeve Cockrell, Sian Saha, Harriet Noble, Kevin O’Reilly, Maria Depante, Anna Broderick, Marianette Anne Axalan, Burt Vergara, Reena Mehta (Co-PI)

**Poole**

Henrik Reschrieter (PI), Sarah Patch, Julie Camsooksai, Sarah Jenkins, Madga Pomichowska, Ken Power, Spike Briggs, Elizabeth Woodward, Christopher Loew, James Bromilow, James Keegan, Matthew Taylor, Emma Langridge, Dinesh Kulandhaisamy, Saah Savage, Yasmin de’Ath, Charlotte Humphrey, Sue Roffe, Matthew Bayliss, Leanne Bartlett

**Royal Marsden**

Richard Gordon-Williams (PI), Kate Tatham (previous PI), Sam Smith, Isabel Noris, Sharjeel Tahir, Emma Yates, Shivali Patel, Tanith Westerman, Sekina Bakare, Hugh Furness, Emma Hunt, Reyhaneh Sadegh Zadeh, Maria Khan, William Sherwood, Claudio Addari, Roshni Manex, Nicole Whitehead, Fred Wilson, Luke Edwards, Kshiteeja Nalk, Sophie Biddle, Suzannah Lant, Francesca Holden, Shree Voralia, Nicola Ocean, Arun Sahni, Prakhar Srilastava, Sultan Iqbal, Shamil Tana, Vishal Venkat Raman, Zoszka Webb, Luke Parker, Arnold Dela Rosa, Miran Kadr, Eleanor Harvey, Ryan Howle, Aatif Husain, Olivia Morley, Sarah Loftus, Jenna Hutchinson, Shaman Jhanji, Ethel Black, David Parkinson, Ravishankar Raobaikady

**Oxford**

Mark Borthwick (PI), Christie James (previous PI), Grace Polley, Neil Davidson, Sally Beer, Paula Hutton, Archana Bashyal, Jean Wilson, Soyamol Mathew, Jung Ryu

**Blackpool**

Jason Cupitt (PI), Gareth Hardy (Co-Inv), Leonie Benham, Robert Downes

**Leicester**

Neil Flint (PI), Michael Little (Co-PI), Ravindra Pochiraju, Prematie Andreou, Dawn Hales, Jessica Hailstone, Megha Mathews

**Harrogate**

Martin Huntley (PI), Lorraine Stephenson, Jacqui Hussey, Hao-Ern Tan, Simon Holbrook, Hayley Kemp, David Earl

**Musgrove Park**

Richard Innes (PI), Benjamin Plumb (Co-PI), Patricia Doble, Rebecca Purnell, Ashly Thomas

**Lewisham**

Muhammad Hamza Noor (PI), Waqas Khaliq (previous PI) Micheal Jennings, Bernd Oliver Rose, Rosaleeta Reece-Anthony, Sagira Khatun, Samantha Dickinson, Jayson Clarke, Charlie Cox, Adam Longley, Tariq Ali, Babita Gurung, Mohamed Moubarak

**St George’s**

Alan Williams (PI), Jonathan Ball (Previous PI), Susannah Leaver, Sarah Farnell-Ward, Maria Thanasi, Shreeja Dangol, Vince Ventura, Massimiliano Valcher, Christine Sicat, Nikki Yun, Rebecca Kanu, Maria Maiz Cordoba, Ha Trinh, Karen Lloyd, Romina Pepermans Saluzzio, Lijun Ding, Helen Farrah, Edna Fernandes

**Belfast**

Chris Nutt (PI), Jon Silversides, Danny McAuley, Peter McGuigan, Emmet Major, Elliott Lonsdale, Nerielle Fundano, Kathryn Ward, Christine Turley, Aisling O’Neill, Stephanie Finn, Jackie Green, Erin Collins, Julie McAuley, Jeanette Mills, Chris Wright, Michelle Growcott

**Newcastle**

Iain McCullagh (PI), Stephen Wright, Ian Clement, Jonathan Shelton, Matthew Faulds, Thomas Hellyer, Harriet Morton, Christopher Pollard, Christopher White, Leigh Dunn, Verity Calder, Susan Taylor, Pamela Garcia, Benjamin Brown, James Savage, Maite Babio-Galan, Kimberley Webster, Tessa Wilkinson, Arti Gulati, Tara Shrestha, Carole Hays, Lauren Butler, Fatima Simoes, Margaret McNeil, Ian Storey

**Leeds**

Simon Whiteley (PI), Elizabeth Wilby, Susan Trott, Sarah Watts, Shailamma Mathew, Sheila Salada, Adam Neep, Nora Youngs, Clare Howcroft, Matthew Powell, Michael Adlam, Elankumaran Paramasivam, Zoe Friar

**Imperial**

David Antcliffe (PI), Stephen Brett (PI), Anthony Gordon (PI), Dorota Banach, Roceld Rojo, Sonia Sousa Arias, Ziortza Fernandez de Pinedo Artaraz, Phoebe Coghlan, Amal Mohammed, Eleanor Jepson, Jenny Wong, Anita Tamang Gurung, Caoimhe O’Dwyer, Sara Perez Guillotin, Maie Templeton

**Manchester**

James Hanison (PI), Jonathan Bannard-Smith, Daniel Conway, Shoneen Abbas, Mohamad Aly, Stephen Benington, Teh Eng Hean, Daniel Hayley, Ellen McGuckin, Andrew Martin, Thomas Morris, William Musselbrook, Bhaskar Narayan, Thomas Wright, Chris Wheeler, Melanie Barker, Richard Clark, Emma Connaughton, Rose Jama**,** Deborah Paripoorani, Rachael Quayle, Anila Sukumaran, Charlotte Taylor, Megan Balmer, Saejohn Lingeswaran**,** Lauren Edmunds, Katharine Wylie

**Birmingham Heartlands**

Andrew Owen (PI), Gavin Perkins (previous PI), Sean Munnelly, Daniel Park, Jo Gresty, Ellie Reeves, Celina Maliaykal, Teresa Melody

**Cambridge**

Jacobus Preller (PI), Petra Polgarova, Cristina Bravoelvira, Sofia Teixeira, James Varley, Sapna Sharma Hajela, Kay Elston, Siobhan Campbell, Meike Keil, Muhammad Elbehery, Jocelyn Marshall, Susan Stevenson, Andrew Conway Morris, Prasad Gogineni Venkateskara

**Russells Hall**

Michael Reay (PI), Karen Reid, Rebecca Brown, Chinenyenwa Amareihe, Elliot Yates, Jia Luen Goh, Edward Jones, Aamer Mughal

**UCLH**

David Brealey (PI), Niall MacCallum, Samuel Clark, Deborah Smyth, Georgia Bercades, Ingrid Hass, Gladys Martir, Jung Ryu, Anna Reyes, Maria Alexandra Zapata Martinez, Laura Gallagher, Chi Yee Chung

**Medway**

Graeme Sanders (PI), Vipal Chawla, Namrata Maheshwari, Tessa Glazebrook, Hollie Angel, Rebecca Squires, Hayley Dolan, Christopher Donnelly, Lucy Mires, Robert Musalagani, Suzanne Williams

**King’s Lynn**

Robin Heij (PI), Peter Young, Mark Blunt, Gayathri Wijewardena, John Gibson, Aricsa Mariya Joshy

**Bristol Royal**

Jeremy Bewley (PI), Kieron Rooney, Katie Sweet, Kim Wright, Lisa Grimmer, Denise Webster, Casandra Bazan Lacerot, Rachel Shiel, Eva Maria Hernandez Morano, Christina Coleman, Eleanor Daniel, Oluwatosin Komolafe, Josephine Bonnici, Linda Pipira, Rebekah Johnson, Anna Chillingworth, Ya-Hui Liang, Georgia Efford, Angeliki Kolovou, George Davies, Zoe Garland, Bethany Gumbrill, Ivan Collin, Matthew Gibbins, Thomas Brougham, Agnieszka Skorko

**Queen’s Nottingham**

Dan Harvey (PI), William Phipps, Kathryn Harrold, Nick Plummer, Ben Lowe, Paul James, Sara Ahmed, Rukmini Ghosh, Omer Mohamed, Tanushree Santra, James Shilston, Andrew Russell, Viresh Patel, Upasana Topiwala, Habideen Bello, Julia Sampson, Lucy Ryan, Cecilia Peters, Megan Meredith, Louise Conner (Now Hughes), Lucy Morris, Amy Clark, Alice Baddeley, Lisa Mcloughlin, Cate Walton, Treesa Joseph, Anju Thomas, Sophie Lubbock, David Ford, Alexandra McCoy, Tony N’Dungu

**Royal Liverpool**

Ingeborg Welters (PI), Vinoth Sankar, Alicia Waite, Brian Johnston, David Shaw, Vicki Waugh, Karen Williams, Maria Lopez Martinez, Maria Norris, Maria Arra Carlota Mahiya, Jamie Fernandez Roman, Jin-Xi. Yuan, Silvia Manes, Caitlin Lythgoe, Ibrahim Almafreji, Josh Colfar, Laura Medhurst, Stephanie Beresford, Sofia Farina, Lema Imam, Syamlam Ali, Zachary Thomas, Francesca Bold, Edward Hughes, Katherine Hodson, Aleem Morenikeji, Daniel Watkin

**Aneurin Bevin**

Tamas Szakmany (PI), Amy Cardwell, Anne Frawley

**Guys & St Thomas**

Marlies Ostermann (PI), Gillian Radcliffe, Nicholas Barrett, Simon Sparkes, Adam Woodman-Bailey, Eirini Kosifidou, Aneta Bociek, Ellie Hendrie, Rosario Lim, Fabiola D’Amato, Sarah Fordyce, Benjie Cendreda, Kyma Morera Vas, Jacqueline Pan, Christopher Meddings, Vladimir Milic, Mike Barker, Jennifer Owusu-Afriyie, Carolin Engelhard

**Queen Elizabeth University Hospital, Glasgow**

Malcolm Sim (PI), Richard Appleton (previous PI), Maximilian Ralston, Andrew Arnott, Steven Henderson, Izabela Orlikowska, Sophie Kennedy-Hay

**Ulster**

Christopher Murray (PI), Matthew Devine (previous PI), Padraig Headley, John McCaffrey, Daniel Donnelly, Richard Young, Samantha Hagan, Victoria Adell, Elizabeth Murphy

**Dumfries & Galloway**

Alasdair Hay (PI), Jian Quek, Stephen Wilson, Catherine Jardine

**Wye Valley**

Mark Forrest (PI), Emma Collins, Miqdad Ibrahim, Mark Wheeley, Mostafa Kodous, Mathew Blake, Victoria Lacey, Michael Eager, Robin Jootun, Janine Birch
